# Identifying key features for determining the patterns of patients with functional dyspepsia using machine learning

**DOI:** 10.3389/fphys.2025.1658866

**Published:** 2025-10-10

**Authors:** Heeyoung Moon, Da-Eun Yoon, Junsuk Kim, Younkuk Choi, Heekyung Kim, In-Seon Lee, Younbyoung Chae

**Affiliations:** ^1^ Acupuncture and Meridian Science Research Center, Kyung Hee University, Seoul, Republic of Korea; ^2^ Department of Meridian and Acupoints, College of Korean Medicine, Semyung University, Jecheon, Republic of Korea; ^3^ School of Information Convergence, Kwangwoon University, Seoul, Republic of Korea; ^4^ Department of Clinical Research Design and Evaluation, Samsung Advanced Institute of Health Sciences and Technology, Sungkyunkwan University, Seoul, Republic of Korea; ^5^ Gangnam-Shingwang ECM Clinic, Seoul, Republic of Korea; ^6^ Yebon ECM Clinic, Seoul, Republic of Korea

**Keywords:** pattern identification, functional dyspepsia, supervised learning, feature extraction, unsupervised learning

## Abstract

**Background and aims:**

Pattern identification (PI) provides a basis for understanding disease symptoms and signs. The aims of this study are to extract features for identifying conventional PI types from the questionnaire data of patients with functional dyspepsia (FD) through supervised learning methods, and to compare them with another set of features for novel PI types identified with unsupervised learning.

**Methods:**

In total, 153 patients with FD were invited to complete the Standardized Tool for Pattern Identification of Functional Dyspepsia (STPI-FD) questionnaire. Supervised analysis using support vector machine (SVM) was conducted to extract the most discriminative features. For unsupervised analysis, t-distributed stochastic neighbor embedding (t-SNE) and k-means clustering were applied to detect novel subgroups, and independent-samples t-tests were performed to identify distinguishing features between clusters.

**Results:**

The SVM identified loss of appetite, flank discomfort, abdominal bloating or gurgling, and pale or yellowish complexion as the most discriminative features. Unsupervised clustering revealed four distinct patient subgroups with differing predominant symptom profiles, such as systemic symptoms, upper abdominal symptoms, changed bowel movement, and nausea/vomiting.

**Conclusion:**

Through supervised learning, we identified the most important features for PI. Additionally, we proposed a novel unsupervised learning approach for identifying patterns from the patient data. This study could facilitate clinical decision making as it pertains to patients with FD.

## 1 Introduction

Functional dyspepsia (FD), one of the most common disorders of gut-brain interaction (DGBI), has a significant impact on the quality and activities of daily life. It is characterized by the presence of one or more dyspeptic symptoms such as postprandial fullness, early satiation, epigastric pain, and epigastric burning (Tack et al., 2006). FD is also characterized by relapse and remission, imposing a high burden on healthcare systems and often remaining unidentified even after routine clinical treatment and evaluation (Talley and Ford, 2015; Chuah et al., 2022). Although conventional treatments such as proton pump inhibitors, prokinetics, and antidepressants are all used, most patients with FD prefer integrative medicine (Rabitti et al., 2021). The complex underlying pathophysiological mechanisms are responsible for the difficulty in curing FD.

Pattern identification (PI) is used in traditional East Asian medicine to help diagnose patients with various diseases (Alraek, 2014). PI provides a basis for holistic, tailored, patient-centered treatment by considering environmental, psychosocial, and individual factors (Robinson et al., 2019). In clinical practice, this process is crucial for providing the personalized treatment to patients, particularly those with functional disorders with complex pathophysiological basis. In particular, PI can improve our understanding of symptoms and signs of patients in the context of traditional Korean medicine (Lee et al., 2014). Symptom clusters associated with *qi*-related entities or structural-functional systems, such as the viscera and bowels (*zangfu*), underlie the standardized, conventional patterns identified by PI analysis (Birch et al., 2020). In the clinical practice guidelines of Korean medicine for FD, which were released in 2021 by the National Institute for Korean Medicine Development, six standardized FD patterns are mentioned.

Various studies on PI are currently in progress, including a database study on traditional PI and a study taking a novel approach to syndrome differentiation based on intragroup similarities in real-world data (Lee and Chae, 2024). Chen *et al.* searched the literature for relevant traditional Chinese medicine studies on Parkinson’s disease and conducted analyses to identify the most frequent and important patterns of symptoms (Chen et al., 2017). Park *et al.* analyzed the questionnaire data of patients with allergic rhinitis and evaluated the decision-making process of traditional Korean medicine doctors according to their explicit and implicit knowledge (Park et al., 2022). With an emphasis on the importance of the reproducibility of PI data, Lee *et al.* proposed a deep learning-based decision-making model using a *k*-means clustering algorithm and applied it to the cross-sectional data of patients with sleep disturbance (Lee et al., 2022). Finally, with consideration of the multidimensional nature of traditional Chinese medicine data, Ho *et al.* conducted a latent tree analysis of data from patients with FD and reported score-based rules for pattern differentiation (Ho et al., 2022a; Ho et al., 2022b). Throughout these previous studies, it can be inferred that PI types can be defined in two ways; one through a conventional and theoretical approach, and the other through modern data science. However, despite these previous studies, there is still a need to compare the subgroupings of patients with FD derived from traditional theory versus data-driven approach applied to clinical data, and it is also necessary to standardize the PI process. Without such comparisons, reliance on a single analytical approach may limit the understanding of how different strategies converge or diverge in identifying clinically meaningful subgroups.

In the context of complex and multidimensional data models, such as clinical data, the application of machine learning methods proves invaluable. Machine learning is primarily divided into two categories: supervised and unsupervised learning. Supervised learning is particularly useful when clinical codes are combined with free-text notes that serve as class labels, while unsupervised learning is effective for analyzing data without annotations (Spasic and Nenadic, 2020). Therefore, it can be inferred that supervised learning methods are applicable to patient data with theoretical labeling. Conversely, unsupervised learning methods do not require data annotation, allowing for observations based on data-driven clustering. In this study, we extracted features from the FD patient-reported questionnaire data using both supervised and unsupervised learning algorithms to differentiate patient subgroups.

## 2 Methods

### 2.1 Study design

This multicenter, prospective, observational registry study was conducted in 15 traditional medical clinics located in the Republic of Korea and managed by Korean medical doctors with ≥5 years of clinical experience. The enrolled patients underwent up to three regular assessments during the treatment period. The patients were provided with a thorough explanation of the study process and were requested to sign a written informed consent form. The study was approved by the Institutional Review Board of Kyung Hee University (Seoul, Republic of Korea; approval number: KHSIRB-22-074RA), registered with the Clinical Research Information Service of the Korea National Institute of Health (registration number: KCT0008145), and conducted in accordance with the Declaration of Helsinki.

### 2.2 Inclusion and exclusion criteria

Patients aged 18–70 years and diagnosed with FD based on the Rome IV criteria were included in this study. According to the Rome IV criteria, FD is diagnosed when patients present with at least one of the following symptoms: bothersome postprandial fullness, early satiation, epigastric pain, or epigastric burning. These symptoms must have persisted for at least 3 months, with onset at least 6 months before diagnosis, and no structural disease evident on upper endoscopy that could account for the symptoms. Postprandial distress syndrome (PDS) was defined as bothersome postprandial fullness or early satiation on ≥ 3 days per week, and epigastric pain syndrome (EPS) was defined as bothersome epigastric pain or epigastric burning on ≥ 1 day per week (Francis and Zavala, 2024).

After confirming eligibility according to the Rome IV diagnostic criteria, doctors conducted structured interviews to assess the duration of patients’ symptoms and to verify the corresponding FD subtypes. During these interviews, the physicians ascertained whether the patients’ symptoms had persisted for a clinically meaningful period (i.e., ≥3 months, with symptom onset at least 6 months prior, as recommended in the Rome IV criteria) and classified them into PDS, EPS or mixed type based on the patients’ symptoms. The final subtype assignment was cross-checked and recorded in the case report form.

Patients were excluded from the study if they had primary diseases causing dysfunction of the GI tract (e.g., central nervous system disease, neoplastic disease, metabolic disease, or inflammatory disease) or were taking medications compromising GI tract function; had received active treatment (e.g., hospitalization for severe FD symptoms) ≤ 3 months before visiting the outpatient clinic of a center of Korean medicine; or were participating in other studies.

In this study, the patients’ baseline characteristics, normalized questionnaire response data and computed PI labels were analyzed.

### 2.3 Questionnaires

All patients completed the Standardized Tool for Pattern Identification of Functional Dyspepsia questionnaire (STPI-FD) on the day of study registration. This questionnaire consists of 36 questions and uses a five-point Likert response scale (range: 0–4). The questions are distributed among the following 12 categories; stomach symptoms, chest symptoms, oral symptoms, nausea and vomiting, food intake, belching, sighing, complexion, mental state, tiredness, cold/heat state, and defecation. Each patient was assigned to a certain pattern type based on their questionnaire responses.

The STPI-FD covers FD symptoms ranging from the typical pathological GI symptoms to secondary “holistic” symptoms. This questionnaire was developed based on 93 clinical South Korean and Chinese studies about the symptoms of FD, with the Delphi method applied to identify the most important symptoms and patterns. The weights reflect the importance of specific symptoms for determining the patterns (Ha et al., 2024). In clinical practice, doctors of traditional Korean medicine ask patients not only about their main symptoms but also about their systemic symptom pattern. We considered the STPI-FD to be the most appropriate instrument for assessing the basic knowledge of doctors of traditional Korean medicine, and exploring how clinical diagnoses are made according to patients’ clinical features.

Patients also completed a number of additional questionnaires. The severity of symptoms was evaluated using the Korean version of the Nepean Dyspepsia Index (NDI-K) (Talley et al., 1999), which assesses the frequency, seriousness, and subjective perceptions of 15 representative symptoms of FD. A functional dyspepsia-related quality of life (FD-QoL) questionnaire was used to assess changes in quality of life in the domains of nutrition, vitality, emotions and social function (Lee et al., 2006).

### 2.4 Feature selection for determining patterns of functional dyspepsia symptoms based on supervised learning

Based on their STPI-FD data, patients were assigned to one of six patterns defined in the clinical practice guidelines of traditional Korean medicine for FD: spleen and stomach deficiency and cold type (PI1), spleen deficiency with *qi* stagnation (PI2), liver-stomach disharmony (PI3), tangled cold and heat (PI4), dampness and heat in the spleen and stomach (PI5), or food retention disorder (PI6).

The raw STPI-FD data were subjected to *z*-score normalization. This process was implemented to reduce bias associated with patients’ subjective perceptions of the severity of their symptoms and emphasize the noticeable symptoms in each patient. Each STPI-FD item corresponded to a feature in the patient dataset.

We evaluated five supervised learning algorithms - decision tree model (DT), random forest method (RFM), support vector machine (SVM), extreme gradient boosting (XGBoost), and light gradient boosting machine (LightGBM) - for classifying six patterns of FD. DT consists of attribute nodes linked to subtrees and nodes labeled with classes (Podgorelec et al., 2002). RFM is an ensemble learning technique that constructs multiple decision trees during model training and merges their results to achieve more accurate predictions, reducing overfitting by averaging multiple trees (Morales et al., 2024). This method is effective in handling large datasets with high dimensionality (Breiman, 2001). SVM is used for classification that identifies the hyperplane that best separates the data points of different classes in a high-dimensional space, maximizing the differences between classes (Cortes and Vapnik, 1995). XGBoost is a scalable tree boosting system in which weak learners can be ensembled iteratively into a strong predictor when the loss function is minimized (Chen and Guestrin, 2016). LightGBM is a relatively modern technique with the combination of two novel data sampling and classification methods (exclusive feature bundling and gradient-based one-side sampling), thus making the process of data scanning, sampling and clustering faster and more accurate (Ke et al., 2017).

The dataset was split into a stratified training set (80%) and test set (20%) to preserve the imbalanced class distribution. Model development and hyperparameter tuning were performed using 5-fold cross-validation across five algorithms. The primary performance metric was balanced accuracy (Brodersen et al., 2010), the mean of recall and specificity, chosen to account for potential class imbalance in the dataset. The final model was then refit on the entire training set and evaluated once on the independent test set.

**FIGURE 1:**
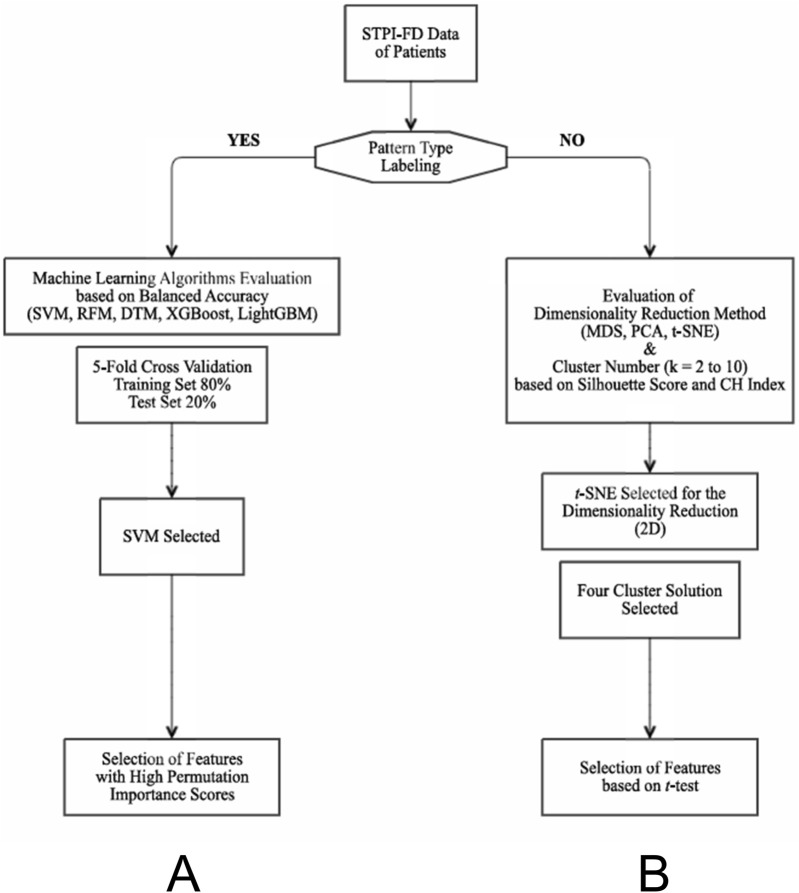
Flow charts of the machine learning algorithms applied in this study. **(A)** Flow chart for the supervised learning method. Feature selection based on balanced importance scores was followed by the evaluation of the most appropriate machine learning algorithm. **(B)** Flow chart for the unsupervised learning method. The most appropriate dimensionality reduction method and the optimal number of subgroups were determined by comparing the silhouette scores and CH index of three representative dimensionality reduction methods. DTM, decision tree model; MDS, multidimensional scaling; PCA, principal component analysis; RFM, random forest method; STPI-FD, Standardized Tool for Pattern Identification of Functional Dyspepsia questionnaire; SVM, support vector machine; *t*-SNE, *t*-distributed stochastic neighbor embedding; CH index, Calinski-Harabasz index.

Finally, feature importance was assessed using impurity- or gain-based measures for tree-based models (DT, RF, XGBoost, and LightGBM) and permutation importance on the test set for all algorithms, including SVM (Figure 1A).

### 2.5 Dimensionality reduction and cluster analysis based on unsupervised learning

Data-driven subgroups were defined through dimensionality reduction. To choose the most appropriate method, we compared the silhouette scores and Calinski-Harabasz (CH) index of three representative dimensionality reduction methods: principal component analysis (PCA), multidimensional scaling (MDS), and *t*-distributed stochastic neighbor embedding (*t*-SNE). PCA is a linear dimensionality reduction method that transforms the data into a new coordinate system, yielding principal components. With PCA, the original variability in the data is retained while reducing the dimensionality (Oshternian et al., 2024). MDS positions datapoints in a low-dimensional space such that the distances between data points are preserved; this makes it useful for determining and representing the similarity of data points (Borg and Groenen, 2007). *t*-SNE is a nonlinear dimensionality reduction method that yields joint probabilities and minimizes Kullback–Leibler divergence between low- and high-dimensional data (Agis and Pozo, 2019; Melit Devassy et al., 2020).

*K*-means clustering was performed for the selection of appropriate number of data-driven clusters. The number of possible clusters (*k*) ranged from two to ten. This was followed by evaluation of clustering performance based on the silhouette scores, which range from −1 to 1; the closer together the participants within the same group, and the farther apart those in different groups, the higher the score. A negative silhouette score indicates poor clustering performance (Leng et al., 2022). CH index, which is the sum of inter-cluster and intra-cluster dispersion (Aik et al., 2023), was also evaluated for the performance. To identify the most appropriate dimensional reduction method and determine the optimal number of subgroups, we compared the clustering performance among the various cluster solutions.

After determining the appropriate dimensionality reduction method and optimal number of clusters, we characterized each novel cluster by identifying features in which each cluster significantly differed from the remaining clusters. Independent *t*-test was performed for each comparison, with significance defined as *p* < 0.05 and effect size (Cohen’s d) ≥ 0.8. (Figure 1B).

### 2.6 Statistical analysis

All analyses were performed using Python (v3.11.13) with scikit-learn (v1.6.1), XGBoost (v3.0.4), and LightGBM (v4.6.0). Figures including bar graph and scatter plot were generated in Python, while heatmap was additionally produced using Orange software (v3.38.1).

## 3 Results

### 3.1 Baseline patient characteristics

In total, 153 patients with FD were enrolled between September 2022 and October 2023. Most of the participants were female (83.0%), and their mean age was 45.2 ± 13.3 years. The proportions of patients satisfying the criteria for PDS and EPS were 85.6% and 37.9%, respectively. The mean total NDI-K score at baseline was 52.2 ± 28.4, and the mean total FD-QoL score at baseline was 27.3 ± 18.1 (Table 1).

**TABLE 1:** Baseline characteristics of the patients.

Characteristics		n (%) or mean ± standard deviation
Sex	Male	26 (17.0%)
	Female	127 (83.0%)
Age (y)		45.2 ± 13.3
BMI		21.6 ± 3.3
NDI-K score		52.2 ± 28.4
FD-QoL score		27.3 ± 18.1
Duration of FD (y)		8.9 ± 12.6
FD subtypes	PDS	95 (62.1%)
	EPS	22 (14.4%)
	PDS and EPS	36 (23.5%)
Comorbidity	IBS	47 (30.7%)
Education level	College and above	120 (78.4%)
	High school	29 (19.0%)
	Other	4 (2.6%)
Marital status	Married	94 (61.4%)
	Unmarried	55 (35.9%)
	Others	4 (2.6%)

BMI, body mass index; EPS, epigastric pain syndrome; FD, functional dyspepsia; FD-QoL, functional dyspepsia-related quality of life questionnaire; NDI-K, korean version of the nepean dyspepsia index; PDS, postprandial distress syndrome; IBS, irritable bowel syndrome.

The numbers of patients classified as PI1, PI5, PI6, PI2, PI4, and PI3 were 79, 22, 19, 15, 11 and 7, respectively in order (Supplementary Material).

### 3.2 Key features for conventional pattern identification using supervised learning

Balanced accuracy was highest for the SVM (0.67), followed by the LightGBM (0.51), DTM (0.48), RFM (0.46) and XGBoost (0.34). The baseline accuracy for all algorithms according to the “zero rate classifier” was 0.17. The SVM was used to identify key features in the labeled dataset by permutation importance score.

**FIGURE 2:**
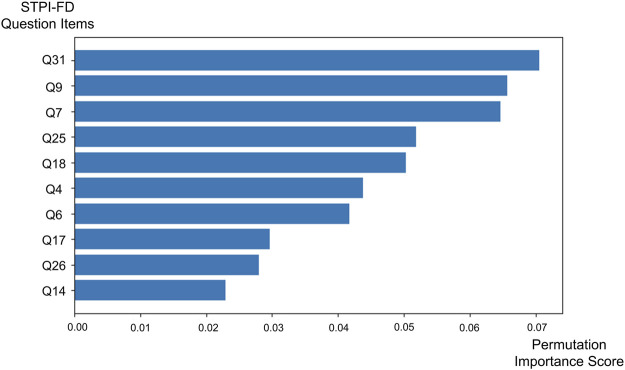
Bar chart showing the top 10 permutation importance scores of the Standardized Tool for Pattern Identification of Functional Dyspepsia questionnaire items obtained via the support vector machine.

The features with the highest permutation importance scores for determining FD symptom patterns were “My pain decreases when my abdomen is warmed or massaged” (STPI-FD question 31; permutation importance score = 0.070 ± 0.037), “My flank feels bloated or painful” (STPI-FD question 9; permutation importance score = 0.066 ± 0.086), “My stomach feels bloated and gurgles” (STPI-FD question 7; permutation importance score = 0.065 ± 0.033), “My complexion is pale and sometimes turns yellow” (STPI-FD question 25; permutation importance score = 0.052 ± 0.051) and “I do not feel like eating” (STPI-FD question 18; permutation importance score = 0.050 ± 0.037) (Figure 2).

### 3.3 Key features for data-driven pattern identification using unsupervised learning

**FIGURE 3:**
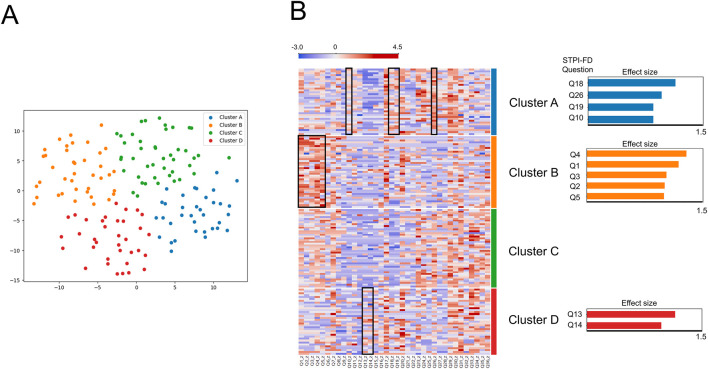
**(A)** Distribution of patients across four novel subgroups. Cluster A (35 blue dots, mainly on the right side); Cluster B (39 orange dots, mainly on the left side); Cluster C (44 green dots, mainly on the upper side); Cluster D (35 red dots, mainly on the lower side). **(B)** Heatmap of the *z*-score normalized Standardized Tool for Pattern Identification of Functional Dyspepsia data of 153 patients: comparison between Cluster A, B, C and D. Questionnaire items highlighted with black squares indicate significantly higher scores in the respective cluster. The highlighted items are additionally depicted as bar charts beside Clusters A, B, and D. Since Cluster C did not show any items exceeding the threshold (effect size 0.8), no bar chart is presented for this cluster.

Comparing all possible clustering and dimensionality reduction solutions, the visualization of data with four clusters in two dimensions yielded the best performance (silhouette score = 0.40, Calinski–Harabasz index = 148.3). The patient distribution across Clusters A to D was 35, 39, 44, and 35, respectively (Figure 3A).

Cluster A showed significantly higher scores in STPI-FD question 26 (“I feel mentally exhausted and my whole body is weak”), 18 (“I do not feel like eating”), 10 (“I have no appetite, but my mouth is not dry”) and 19 (“I have no appetite and feel tightness after eating”) (all *p* < 0.001; Cohen’s d = 0.96, 1.14, 0.85 and 0.85, respectively). Cluster B showed significantly higher scores in STPI-FD question 4 (“My upper abdomen feels heavy and painful, like indigestion, and the pain worsens when pressed”), 1 (“My upper abdomen feels tight and occasionally mildly painful”), 5 (“My chest and upper abdomen feel sore or painful or hungry, making me uncomfortable”), 3 (“My upper abdomen feels bloated and occasionally painful”) and 2 (“My upper abdomen feels tight and occasionally severely painful”) (all *p* < 0.001; Cohen’s d = 1.29, 1.19, 1.00, 1.03 and 1.01, respectively). Cluster C did not show significantly higher scores compared with other clusters. Cluster D showed significantly higher scores in STPI-FD question 13 (“I sometimes feel nauseous or vomit”) and 14 (“I sometimes feel nauseous or vomit, and the symptoms reduce after vomiting”) (both *p* < 0.001; Cohen’s d = 1.15 and 0.97). (Figure 3B).

## 4 Discussion

We conducted a prospective observational study in which FD patient-reported questionnaire data were analyzed using various machine learning algorithms. Through supervised learning, we identified the most important features for assigning patients to six different PI types. We also applied unsupervised learning technique for revealing novel subtypes and identified several commonalities and differences in the PI results obtained using the two approaches.

In this study, we first categorized patients with FD on the basis of their STPI-FD scores using conventional methods. PI1 was the most prevalent pattern (51.6%), followed by PI5 (14.4%), PI6 (12.4%), PI2 (9.8%), PI4 (7.2%), and PI3 (4.6%). The distribution of the patterns of the patients with FD in this study were similar to those in a previous study, in which the PI1 pattern was the most prevalent (51.6%) among 95 patients with FD followed by PI5 (14.7%), PI4 (13.7%), PI2 (9.5%), PI3 (7.4%), and PI6 (3.2%) (Ha et al., 2024). Thus, the PI1 (51.6% and 51.6%, respectively) and PI5 (14.4% and 14.7%, respectively) patterns were the most prevalent in both primary care clinics and hospitals. Practitioners can use PI to diagnose patients with FD and develop treatment plans.

At first, we performed supervised learning to identify the most important features for distinguishing symptom patterns based on the questionnaire responses of patients with FD. The main features identified by the SVM model according to their permutation importance scores were related to abdominal and flank discomfort, change in complexion and loss of appetite. The aim of supervised learning was to identify the most important features or explanatory variables from among many candidates in real-world, high-dimensional clinical datasets (Huang et al., 2023).

The “decrease in abdominal pain when warmed or massaged” and “pale or yellow complexion” features, associated with PI1, had the fourth- and fifth-largest weights among the eight items in the corresponding calculation formula. The “painful feeling in flank” and “loss of appetite” feature, associated with PI3, had the highest and sixth-highest weights among the eleven items in the corresponding calculation formula. The “loss of appetite” and “bloated feeling in stomach” feature, associated with PI4, had top two weight among nine items in the corresponding calculation formula. The “loss of appetite” feature was also associated with PI6, ranking third in weight among the seven corresponding items.

Based on patient-reported questionnaire data, patients classified as PI1 can be characterized by alleviation of abdominal discomfort in response to warm stimuli or massage, as well as by changes in complexion. The classification of patients as PI3 was mainly characterized by flank discomfort, a finding that corresponds well with expert clinical opinions. Patient classification as PI4 was characterized by bloated feeling in stomach. Finally, loss of appetite was a common feature across multiple PI types based on patient-reported data (PI3, PI4 and PI6). Therefore, this feature should be interpreted with caution in syndrome differentiation, as it overlaps across several PI categories. Using machine learning and symptom-related questionnaire data, our study shed light on the most important symptoms for practitioners to be aware of for diagnosing and developing treatment plans for FD.

We also conducted unsupervised learning as a novel patient classification approach. *K*-means clustering revealed that patients with FD can be divided into four distinct subgroups. To illustrate the distinct characteristics of these subgroups, we initially focus on a comparison between two of them; one characterized by “systemic comorbidity predominant cluster” (Cluster A) and another by “gastrointestinal symptom predominant cluster” (Cluster B). Questions (i.e., features) with scores that differed significantly between the subgroups were considered crucial for distinguishing patterns. Scores for questions related to fatigue, loss of appetite, and lack of energy were significantly higher in the first cluster. In contrast, all features related to upper abdominal discomfort had significantly higher scores in the second cluster. In line with the visualized plot in this study, Cluster A and Cluster B demonstrated distinct tendencies along the axis, which may aid in clinical differentiation between systemic comorbidity and upper abdominal symptom.

Unlike these two contrasting clusters, the remaining two clusters (Clusters C and D) are discussed separately, as each demonstrates unique characteristics. Cluster C did not demonstrate any features that met the statistical thresholds. However, patients in this cluster tended to report comparatively higher scores on questions 33 to 36, suggesting a tendency toward decreased function of the bowel movement. Meanwhile, Cluster D was characterized by significantly higher scores in nausea- and vomiting-related items. This indicates that, even within a cohort of patients diagnosed with FD, distinct subgroups can be identified based on the predominant symptoms reported. Such data-driven clustering highlights the heterogeneity of symptom presentation and provides a potential framework for more individualized approaches to syndrome differentiation and clinical management.

Unsupervised machine learning is increasingly being applied in studies of traditional Korean medicine, especially for PI. The goal of unsupervised learning was to divide patients into subgroups characterized by within-group homogeneity and between-group heterogeneity (Huang et al., 2023). PI theories can be distinguished according to whether they are focused on the main pathological symptoms of a disease or the overall health status of individual patients (Birch et al., 2020). Comprehensive data from traditional Korean medicine clinics provide insight not only into the main pathological symptoms of a disease but also the characteristics of individual patients.

Throughout this process, we aimed to compare two branches of categorization process. At first part, by utilizing supervised learning methods, we could compare commonality and specificity between classical labeling based on experts’ opinion and data analysis from the clinics with label annotation in the aspect of feature selection. At second part, by conducting unsupervised learning methods, we could extract the novel subgroups by lowering dimensionality of clinical data. Overall, we could select important features for assorting PI types and organize four novel subgroups based on data-driven analysis without classical labeling of PI.

Machine learning algorithms can improve our understanding of how traditional Korean medical doctors learn by extracting high-dimensional information from clinical data (Bae et al., 2021). It is important to consider the complexity of the decision-making process in traditional Korean medical clinics. Clinical decisions made based on disease symptoms are associated with so-called “branch treatment”, whereas decisions based on PI are associated with “root treatment” (Birch and Alraek, 2014). The features that inform PI include chief complaints and systemic symptom pattern, which tend to be correlated with each other. In the PI process, multicollinearity is eliminated, and the dimensionality of the data is reduced (Lee et al., 2020). Based on the empirical clinical findings of studies on Korean medicine, a step-by-step approach to machine learning will be crucial to refine further the PI process. Furthermore, other analytic approaches, such as latent tree analysis, can also be implemented to generate probabilistic models of the relationships among multiple variables (Zhang et al., 2017). Latent tree analysis was previously applied to the real-world medical data of patients with FD as a novel machine learning approach, to identify the latent variables most important for PI (Ho et al., 2022a; Ho et al., 2022b). However, this method is most suitable for dichotomous data, whereas our dataset comprises of continuous variables (numerical scores). For studies performing advanced PI analyses with the aim of revealing latent layers in datasets of traditional Korean medicine patients, the application of additional data processing methods should be considered.

Several limitations of this study should be acknowledged. First, the patients were unevenly distributed across the different patterns, and the overall sample size was relatively small. These factors may have contributed to the imbalance in cluster analysis and limited generalizability, although we implemented stratified splitting and used balanced accuracy as the primary performance metric. In addition, the unequal distribution of patients across PI types reflects the real-world clinical situation, as also noted in previous study on the development of the STPI-FD questionnaire. This constitutes both a limitation for machine learning and an essential characteristic of clinical data. Future studies should further investigate analytical strategies that account for this trait. Second, the lack of external validation limits the direct applicability of our findings to real-world clinical settings. Although we conducted thorough internal validation, this alone cannot fully ensure the robustness or external generalizability of the model. Third, the STPI-FD data rely solely on patients’ self-reporting. Other examinations such as pulse and tongue diagnosis, which are important for making decisions in traditional Korean medicine, were not included in the analysis. While reliance on self-reported questionnaires inevitably provokes a lack of objectivity, it was essential for labeling conventional PI types and comparing emphasized features between patterns in this study. However, to further strengthen the robustness and applicability of the findings, the integration of additional data, including objective biomarkers related to FD, such as detection of duodenal images (Mihara et al., 2025). Fourth, the comorbidities and potential confounding factors of FD were not adequately reported. In clinical practice, symptom overlap among DGBI occurs frequently (Fairlie et al., 2023). However, in this study, only comorbidity with IBS was reported. Other confounding factors of FD such as *H.pylori* infection and its eradication (Ford et al., 2022) were not considered in this study. Therefore, the pathological implications of this study should be interpreted with caution. Finally, this study did not conduct an association analysis with treatment response. Future studies should incorporate this factor, such as the effect of acupuncture treatment on FD, to broaden the application of artificial intelligence and allow for a refined reinterpretation of PI.

In conclusion, this study analyzed FD patient-reported data from clinics, for the first time, in the context of traditional Korean medicine, and applied machine learning methods to detect features important for PI. Our results could inform decision making as it pertains to assigning patients with FD to subgroups based on PI.

## Data Availability

The original contributions presented in the study are included in the article/Supplementary Material, further inquiries can be directed to the corresponding author.

## References

[B1] Agis D. Pozo F. (2019). A frequency-based approach for the detection and classification of structural changes using t-SNE. Sensors (Basel) 19, 5097. 10.3390/s19235097 31766460 PMC6928785

[B2] Aik L. E. Choon T. W. Abu M. S. (2023). “K-means algorithm based on flower pollination algorithm and Calinski-Harabasz Index,” in Journal of physics: conference series (Bristol, United Kingdom: IOP Publishing).

[B3] Alraek T. (2014). Designing clinical studies that take into account traditional East Asian medicine's systems and methods - with focus on pattern identification. Chin. J. Integr. Med. 20, 332–335. 10.1007/s11655-014-1807-5 24788085

[B4] Bae H. Lee S. Lee C. Y. Kim C. E. (2021). A novel framework for understanding the pattern identification of traditional asian medicine from the machine learning perspective. Front. Med. (Lausanne) 8, 763533. 10.3389/fmed.2021.763533 35186965 PMC8853725

[B5] Birch S. Alraek T. (2014). Traditional East Asian medicine: how to understand and approach diagnostic findings and patterns in a modern scientific framework? Chin. J. Integr. Med. 20, 336–340. 10.1007/s11655-014-1809-3 24788086

[B6] Birch S. Alraek T. Bovey M. Lee M. S. Lee J. A. Zaslawski C. (2020). Overview on pattern identification–history, nature and strategies for treating patients: a narrative review. Eur. J. Integr. Med. 35, 101101. 10.1016/j.eujim.2020.101101

[B7] Borg I. Groenen P. J. (2007). Modern multidimensional scaling: theory and applications. Springer Science and Business Media.

[B8] Breiman L. (2001). Random forests. Mach. Learn. 45, 5–32. 10.1023/a:1010933404324

[B9] Brodersen K. H. Ong C. S. Stephan K. E. Buhmann J. M. (2010). “The balanced accuracy and its posterior distribution,” in 2010 20th international conference on pattern recognition: IEEE, 3121–3124.

[B10] Chen T. Guestrin C. (2016). “Xgboost: a scalable tree boosting system,” in Proceedings of the 22nd acm sigkdd international conference on knowledge discovery and data mining, 785–794.

[B11] Chen H. Zhang Z. He J. Teng L. Yuan C. (2017). Traditional Chinese Medicine symptom pattern analysis for Parkinson's disease. J. Tradit. Chin. Med. 37, 688–694. 10.1016/s0254-6272(17)30324-2 32188231

[B12] Chuah K. H. Cheong S. Y. Lim S. Z. Mahadeva S. (2022). Functional dyspepsia leads to more healthcare utilization in secondary care compared with other functional gastrointestinal disorders. J. Dig. Dis. 23, 111–117. 10.1111/1751-2980.13082 35050547

[B13] Cortes C. Vapnik V. (1995). Support-vector networks. Mach. Learn. 20, 273–297. 10.1023/a:1022627411411

[B14] Fairlie T. Shah A. Talley N. J. Chey W. D. Koloski N. Lee Y. Y. (2023). Overlap of disorders of gut–brain interaction: a systematic review and meta-analysis. Lancet Gastroenterology and Hepatology 8, 646–659. 10.1016/S2468-1253(23)00102-4 37211024

[B15] Ford A. C. Tsipotis E. Yuan Y. Leontiadis G. I. Moayyedi P. (2022). Efficacy of Helicobacter pylori eradication therapy for functional dyspepsia: updated systematic review and meta-analysis. Gut 71, 1967–1975. 10.1136/gutjnl-2021-326583 35022266

[B16] Francis P. Zavala S. R. (2024). “Functional dyspepsia,” in StatPearls (Island, FL, USA: StatPearls Publishing). 32119450

[B17] Ha N. Y. Ko S. J. Park J. W. Kim J. (2024). Development of a standard Tool of pattern identification for functional dyspepsia: a cross-sectional study from Korea. Healthc. (Basel) 12, 2331. 10.3390/healthcare12232331 39684953 PMC11641813

[B18] Ho L. Xu Y. Zhang N. L. Ho F. F. Wu I. X. Y. Chen S. (2022a). Quantification of prevalence, clinical characteristics, co-existence, and geographic variations of traditional Chinese medicine diagnostic patterns via latent tree analysis-based differentiation rules among functional dyspepsia patients. Chin. Med. 17, 101. 10.1186/s13020-022-00656-x 36038888 PMC9425972

[B19] Ho L. Zhang N. L. Xu Y. Ho F. F. Wu I. X. Chen S. (2022b). Latent tree analysis for the identification and differentiation of evidence-based Traditional Chinese Medicine diagnostic patterns: a primer for clinicians. Phytomedicine 106, 154392. 10.1016/j.phymed.2022.154392 35994848

[B20] Huang H. Tang Z. Zhang T. Yang B. Song Q. Su J. (2023). Feature selection for unsupervised machine learning. IEEE Int. Conf. Smart Cloud 2023, 164–169. 10.1109/smartcloud58862.2023.00036 38706555 PMC11070246

[B21] Ke G. Meng Q. Finley T. Wang T. Chen W. Ma W. (2017). “Lightgbm: a highly efficient gradient boosting decision tree” in In Advances in Neural Information Processing Systems. (Long Beach, CA, United States: NIPS) , 30.

[B22] Lee Y.-S. Chae Y. (2024). Pattern identification and acupuncture prescriptions based on real-world data using artificial intelligence. East Asian Sci. Technol. Soc. An Int. J. 19, 267–284. 10.1080/18752160.2024.2339657

[B23] Lee E. H. Hahm K. B. Lee J. H. Park J. J. Lee D. H. Kim S. K. (2006). Development and validation of a functional dyspepsia-related quality of life (FD-QOL) scale in South Korea. J. Gastroenterol. Hepatol. 21, 268–274. 10.1111/j.1440-1746.2006.04196.x 16460485

[B24] Lee T. Jung W. M. Lee I. S. Lee Y. S. Lee H. Park H. J. (2014). Data mining of acupoint characteristics from the classical medical text: DongUiBoGam of Korean medicine. Evid. Based Complement. Altern. Med. 2014, 329563. 10.1155/2014/329563 25574179 PMC4276123

[B25] Lee I.-S. Ryu Y. Chae Y. (2020). The principle of acupoint selection based on branch and root treatment. Korean J. Acupunct. 37, 203–208. 10.14406/acu.2020.015

[B26] Lee H. Choi Y. Son B. Lim J. Lee S. Kang J. W. (2022). Deep autoencoder-powered pattern identification of sleep disturbance using multi-site cross-sectional survey data. Front. Med. (Lausanne) 9, 950327. 10.3389/fmed.2022.950327 35966837 PMC9374171

[B27] Leng D. Zheng L. Wen Y. Zhang Y. Wu L. Wang J. (2022). A benchmark study of deep learning-based multi-omics data fusion methods for cancer. Genome Biol. 23, 171. 10.1186/s13059-022-02739-2 35945544 PMC9361561

[B28] Melit Devassy B. George S. Nussbaum P. (2020). Unsupervised clustering of hyperspectral paper data using t-SNE. J. Imaging 6, 29. 10.3390/jimaging6050029 34460731 PMC8321027

[B29] Mihara H. Nanjo S. Motoo I. Ando T. Fujinami H. Yasuda I. (2025). Artificial intelligence model on images of functional dyspepsia. Artif. Intell. Gastrointest. Endosc. 6. 10.37126/aige.v6.i1.105674

[B30] Morales N. Valdes-Munoz E. Gonzalez J. Valenzuela-Hormazabal P. Palma J. M. Galarza C. (2024). Machine learning-driven classification of urease inhibitors leveraging physicochemical properties as effective filter criteria. Int. J. Mol. Sci. 25, 4303. 10.3390/ijms25084303 38673888 PMC11049951

[B31] Oshternian S. R. Loipfinger S. Bhattacharya A. Fehrmann R. S. N. (2024). Exploring combinations of dimensionality reduction, transfer learning, and regularization methods for predicting binary phenotypes with transcriptomic data. BMC Bioinforma. 25, 167. 10.1186/s12859-024-05795-6 38671342 PMC11046904

[B32] Park M. Kim M. H. Park S. Y. Choi I. Kim C. E. (2022). Individualized diagnosis and prescription in traditional medicine: decision-making process analysis and machine learning-based analysis Tool development. Am. J. Chin. Med. 50, 1827–1844. 10.1142/S0192415X2250077X 36056467

[B33] Podgorelec V. Kokol P. Stiglic B. Rozman I. (2002). Decision trees: an overview and their use in medicine. J. Med. Syst. 26, 445–463. 10.1023/a:1016409317640 12182209

[B34] Rabitti S. Giovanardi C. M. Colussi D. (2021). Acupuncture and related therapies for the treatment of gastrointestinal diseases. J. Clin. Gastroenterol. 55, 207–217. 10.1097/MCG.0000000000001455 33116064

[B35] Robinson N. Bovey M. Lee J. A. Zaslawski C. Tian P. Kim T.-H. (2019). How do acupuncture practitioners use pattern identification–an international web-based survey? Eur. J. Integr. Med. 32, 100997. 10.1016/j.eujim.2019.100997

[B36] Spasic I. Nenadic G. (2020). Clinical text data in machine learning: systematic review. JMIR Med. Inf. 8, e17984. 10.2196/17984 32229465 PMC7157505

[B37] Tack J. Talley N. J. Camilleri M. Holtmann G. Hu P. Malagelada J. R. (2006). Functional gastroduodenal disorders. Gastroenterology 130, 1466–1479. 10.1053/j.gastro.2005.11.059 16678560

[B38] Talley N. J. Ford A. C. (2015). Functional dyspepsia. N. Engl. J. Med. 373, 1853–1863. 10.1056/NEJMra1501505 26535514

[B39] Talley N. J. Haque M. Wyeth J. W. Stace N. H. Tytgat G. N. Stanghellini V. (1999). Development of a new dyspepsia impact scale: the Nepean Dyspepsia Index. Aliment. Pharmacol. Ther. 13, 225–235. 10.1046/j.1365-2036.1999.00445.x 10102954

[B40] Zhang N. L. Fu C. Liu T. F. Chen B. X. Poon K. M. Chen P. X. (2017). A data-driven method for syndrome type identification and classification in traditional Chinese medicine. J. Integr. Med. 15, 110–123. 10.1016/S2095-4964(17)60328-5 28285616

