# Supplementary material for: Identifying key features for determining the patterns of patients with functional dyspepsia using machine learning
Source: Front Physiol. 2025 Oct 10;16:1658866. doi: 10.3389/fphys.2025.1658866 (PMC12549581; doi:10.3389/fphys.2025.1658866)
Supplement: Supplementary file 1 [file Supplementaryfile1.doc]

**Appendix.**

**Pattern Identification Types of Patients and Distribution of Baseline Characteristics**

| **Pattern**  **Identification**  **Type** | **Number of**  **Patients (%)** | **Gender** | | **Age** | **NDI-K**  **Score** | **FD-QoL**  **Score** |
| --- | --- | --- | --- | --- | --- | --- |
| PI1 | 79 (51.6%) | Male | 12 | 45.1±14.5 | 53.9±28.1 | 30.6±18.2 |
|  |  | Female | 67 |  |  |  |
| PI2 | 15 (9.8%) | Male | 6 | 44.3±10.3 | 51.3±30.9 | 32.3±21.5 |
|  |  | Female | 9 |  |  |  |
| PI3 | 7 (4.6%) | Male | 0 | 42.1±9.7 | 61.3±43.0 | 25.7±19.9 |
|  |  | Female | 7 |  |  |  |
| PI4 | 11 (7.2%) | Male | 1 | 52.9±10.5 | 42.4±26.5 | 16.4±14.4 |
|  |  | Female | 10 |  |  |  |
| PI5 | 22 (14.4%) | Male | 4 | 44.2±11.9 | 47.9±23.8 | 21.5±14.3 |
|  |  | Female | 18 |  |  |  |
| PI6 | 19 (12.4%) | Male | 3 | 44.7±12.7 | 53.4±25.7 | 22.8±14.0 |
|  |  | Female | 16 |  |  |  |

Data are shown in number (%) or mean ± standard deviation.

PI, pattern identification; PI1[spleen and stomach deficiency and cold]; PI2[spleen deficiency with qi stagnation]; PI3[liver–stomach disharmony]; PI4[tangled cold and heat]; PI5[dampness and heat in the spleen and stomach]; PI6[food retention disorder]
